# NORM dust intake: comparison of personal air sampling and bioassays

**DOI:** 10.1007/s00411-026-01233-8

**Published:** 2026-06-08

**Authors:** Gregory S. Hewson, Martin I. Ralph, Marcus Cattani

**Affiliations:** School of Medical and Health Sciences, Edith Cowan University, 270 Joondalup Drive, Joondalup, Western 6027 Australia

**Keywords:** Thorium, Personal air sampling, Internal dosimetry, Mining, Natural radiation

## Abstract

Intake of naturally occurring radioactive materials (NORM), particularly the inhalation of thorium-bearing dust in mineral sand operations, has traditionally been assessed via personal air sampling (PAS). However, data from bioassay studies suggest that the actual intake determined via PAS may be underestimated. This review integrates thorium intake estimates derived from historical bioassay datasets, such as urine, faeces, lung counting, and thoron-in-breath tests, with historical PAS data. The findings indicated that bioassay-based predictions of intake were consistently greater, with mean bioassay-to-PAS intake ratios ranging from 2.6 to 4.2. The contributing factors to this discrepancy include biokinetic model uncertainties, potential dust sampler bias, the PAS strategy, high variability of exposure within similar exposure groups (SEGs), self-absorption of alpha particles during the radiometric analysis of filtered dust samples, and nonaccounting for respiratory protection. This review suggests improvements to current PAS-based dose assessments, including sampling and analysis protocols. We recommend that PAS be supplemented by targeted bioassay monitoring, where feasible, to improve dose estimation and worker protection in NORM industries.

## Introduction

An accurate assessment of internal radiation dose for workers handling naturally occurring radioactive materials (NORM) is necessary to demonstrate the appropriateness of protection measures and compliance with regulatory requirements, and to indicate if health surveillance is needed or justified. In the Western Australian mineral sand industry (MSI) and other NORM industries, personal air sampling (PAS) for the inhalable dust fraction (0.1–100 μm equivalent aerodynamic diameter) has been the preferred method for monitoring airborne radionuclides, notably members of the thorium and uranium decay chains (IAEA, 2018; GWA, 2010). Thorium intake is of primary interest in the MSI as monazite, a component of mineral sand concentrates, typically contains 5–7% thorium. Uranium is also present in monazite, but typically at 0.2–0.3%.

PAS provides operationally convenient estimates of inhalation exposure; however, determining an accurate intake of radionuclides from PAS relies on the collection and analysis of the relevant fraction of airborne dust, the representativeness of similar exposure groups (SEGs) to which workers are assigned, and use of an appropriate breathing rate for the tasks being undertaken. Furthermore, the conversion of PAS-derived intakes into doses requires assumptions concerning the particle size distribution of inhaled dust, solubility of the dust in the respiratory tract, breathing mode, and knowledge of the radionuclides associated with airborne dust.

Reinterpretation of past bioassay studies using current dosimetric and biokinetic models has recently been conducted in the Western Australian MSI. Updated thorium intake estimates (Bq d^− 1^) from bioassays were obtained across studies involving four different thorium bioassay tests: urine (Hewson et al. 2025b), faeces (Hewson et al. 2025d), and thoron-in-breath and in vivo lung counting (Hewson et al. 2026b). These studies revealed that the mean daily thorium intake estimates from PAS were consistently lower than those from bioassays, raising questions about the reliability of the PAS-based estimates. Owing to the prevalence and reliance on data derived from PAS, such a potential underestimation of intake may translate to ineffective allocation of resources or inadequate worker health protection. An important consideration relates to the manner in which PAS-derived dose estimates are used in future health effect studies.

The International Commission on Radiological Protection (ICRP) indicates a preference for bioassays, where feasible, over PAS when assessing individual radionuclide intake (ICRP 2015). Bioassay measurements provide a direct measure of an individual’s internal radionuclide retention or excretion because the measurement accounts for individual physiology and metabolism and the effectiveness of respiratory protection. Although bioassay techniques are typically costlier and more complex than PAS from the perspective of sample collection, analysis, and interpretation, they may offer greater accuracy, especially for individuals with prolonged and variable exposure histories. Singh and Bertelli (2002) highlight the significant uncertainties associated with both bioassay and PAS techniques and conclude that a combination of PAS and bioassay, using the most conservative estimates, may provide the best protection for workers. A recent literature review found only three published bioassay studies since 2000 relating to exposure to thorium-bearing mineral dust (Hewson et al. 2025a). These studies did not provide an analysis comparing PAS to bioassay, however, one study (Lipsztein et al. 2003) highlighted that there was a lack of correlation between the bioassay results and PAS using cyclone devices (i.e., collecting the respirable fraction of dust).

Differences in radionuclide intake between PAS and bioassays have been observed in other studies. Britcher and Strong (1994) reviewed the use of PAS at two nuclear facilities in the UK and reported a poor correlation between PAS and bioassays. Roberts and Bull (2007) flagged the uncertainty inherent in correlating PAS-derived intake to bioassay measurements following acute exposure events at civil nuclear sites. They attributed the largest uncertainties to include worker breathing patterns and whether the PAS device collects the activity inhaled. Leggett et al. (2022) highlighted that air monitoring data provide unreliable predictors of intake by nuclear plant workers on the basis of discrepancies with bioassay data and concluded that PAS-derived dose estimates should be considered highly uncertain unless there is confidence that PAS data provide representative intakes. Sanchez-Leon et al. (2022) reported that the intake of uranium oxide aerosols by seven workers via urinalysis was approximately threefold higher (range 1.1–4.8) than that via stationary air samplers in the workplace and suggested that the differences observed between workers were likely due to individual physiological characteristics that differed from those assumed for the reference worker in the current ICRP biokinetic model. The cited PAS vs. bioassay comparisons related to studies in nuclear facilities not NORM industries where radionuclides are typically a minor constituent of the mineral dust matrix. Furthermore, activity median aerodynamic diameters (AMAD) in mining and mineral treatment plants have been reported as larger than the ICRP default value of 5 μm for occupational exposure (Dorrian and Bailey 1995; Hewson et al. 2025e).

Van der Steen et al. (2004) report that bioassay techniques are not generally suitable for NORM dust intake owing mainly to insufficient sensitivity. Those authors recommended the use of PAS but highlighted several factors to consider when implementing air sampling strategies, including the collection efficiency of the sampler, and the statistical uncertainties when worker exposures are based on a small number of samples.

The objectives of this study were to summarise the findings from recent reinterpretation of historical thorium bioassay data (Hewson et al. 2025b, d, 2026b) and to investigate the reasons for the differences observed between PAS- and bioassay-derived thorium intake. The earlier studies by the authors addressed specific aspects of the historical bioassay and PAS datasets individually. This present study provides an integrated statistical comparison across all the investigations with a specific emphasis on potential underestimation of PAS-derived intakes in NORM operations. Key methodological issues such as SEG assignment validity, impact of alpha particle self-absorption, biokinetic model sensitivity to changes in key parameters, model uncertainties, and the issue of respiratory protection use were also investigated. The goal was to provide recommendations that may be adopted by regulatory agencies and operators to improve PAS sampling and analysis protocols in NORM industries, including complementing PAS with bioassay measurements, where feasible, to enhance occupational dose assessments.

## Methods

This study reviewed recently published studies on the reinterpretation of past bioassay tests conducted in the 1990s on Western Australian MSI workers. In this study, a reference to thorium refers to ^232^Th unless stated otherwise.

### Mineral sand industry bioassay research

The daily thorium intakes derived from the bioassay tests of MSI workers were compared with those derived from MSI exposure records (i.e., via PAS), as reported in the following three studies:

1. Thorium concentration in 24-hour urine samples collected from 33 workers (Hewson et al. 2025b);
2. Thorium concentration in faecal samples collected over 10-days from 2 workers (Hewson et al. 2025d);
3. Thorium lung burdens estimated via measurements of thoron (^220^Rn) exhaled in breath (TIB) from 207 workers and directly via in vivo lung counting of 19 workers (Hewson et al. 2026b);

The 261 bioassay tests involved 204 workers with exposure periods varying from 1 to 34 years. In the original studies, thorium in 24-hour urine samples was measured by neutron activation analysis (Hewson & Fardy, 1992), daily faecal thorium was measured by inductively coupled mass spectroscopy (Terry et al. 1995), and TIB was measured using the double-filter tube method (Terry et al. 1997).

Studies 1–3 above summarised the bioassay data collected and outlined the methodology used to reinterpret the data via the Taurus internal dosimetry software application (UK Health Security Agency, 2024) which utilises the current ICRP dosimetric and biokinetic models for thorium (ICRP 2015, 2017). The in vivo study provided the mean thoron exhalation rate (3.7 ± 1.2%) for the workers tested during the TIB studies.

## Airborne radioactivity and thorium intake data

The airborne radioactivity data and intake duration (i.e., time employed) used in this study were as reported in the original research, and no changes were made to workers’ historical exposure records. However, when comparing historical bioassay-derived intakes to recorded intakes, reliable PAS data for airborne radioactivity for the MSI workforce were available only from 1985 (i.e. seven years prior to the first bioassay study) (Hewson and Terry 1995). Hence, estimates of intake before this time had significant uncertainty and required broad assumptions regarding potential workers’ exposure to airborne radioactivity concentrations. In contrast, the intakes of workers who commenced in and after 1985 were considered less uncertain because they were based on a substantial number (> 400–500) of yearly PAS measurements performed via regulator-approved protocols (Hewson and Terry 1995). Daily thorium intake was calculated in the original research studies as the cumulative intake of total alpha activity from company exposure records divided by the intake duration at the time of bioassay testing and the number of alpha emissions (*n* = 6) associated with the thorium series in equilibrium.

Table 1 summarises the intake methodology applied between 1959 and 1996, which covered the employment duration of the workers involved in the various bioassay tests. The PAS protocols used by the mineral sand industry have essentially remained unchanged since 1985, except for the type of sample head used to measure inhalable dust (GWA, 2010).

**Table 1:** Thorium intake methodology adopted in past bioassay studies of MSI workers

Intake Period	Thorium intake methodology^a^
1959–1976	Retrospective assessment for mineral separation dry-plant operators based on historical annual volume of monazite production reported to government, an estimated average dust concentration, and a concentration factor for thorium in airborne dust.
1977–1984	Retrospective assessment for dry-plant operators based on industry gravimetric dust concentrations (mg·m^− 3^) recorded by PAS and an assumed specific alpha activity of dust (Bq·mg^− 1^, based on % monazite in feed material). Supervisory and maintenance workers were assigned an intake of one-third the value of dry-plant operators and other workers with occasional exposure in the dry-plant were assigned one-tenth of the value.
1985–1996	Thorium intakes obtained directly from PAS measurements of airborne radioactivity (total alpha activity) concentrations on SEGs as reported by each MSI site. Similar PAS protocols, including dust sampling heads and radiometric analysis, were used by each site.

^a^Based on summaries of historical intake protocols (Hewson 1997; Hewson and Terry 1995).

## Internal dosimetry

**Fig. 1:**
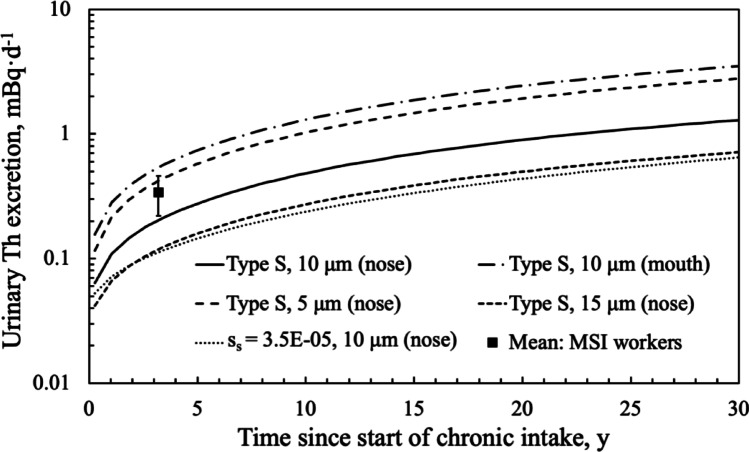
Daily urinary thorium excretion for inhalation of 1 Bq·d^− 1^ thorium as a function of intake duration and different model input parameters. The group mean result from past urine bioassay tests of 26 MSI workers is also shown, with the error bar indicating the 95% confidence interval

**Fig. 2:**
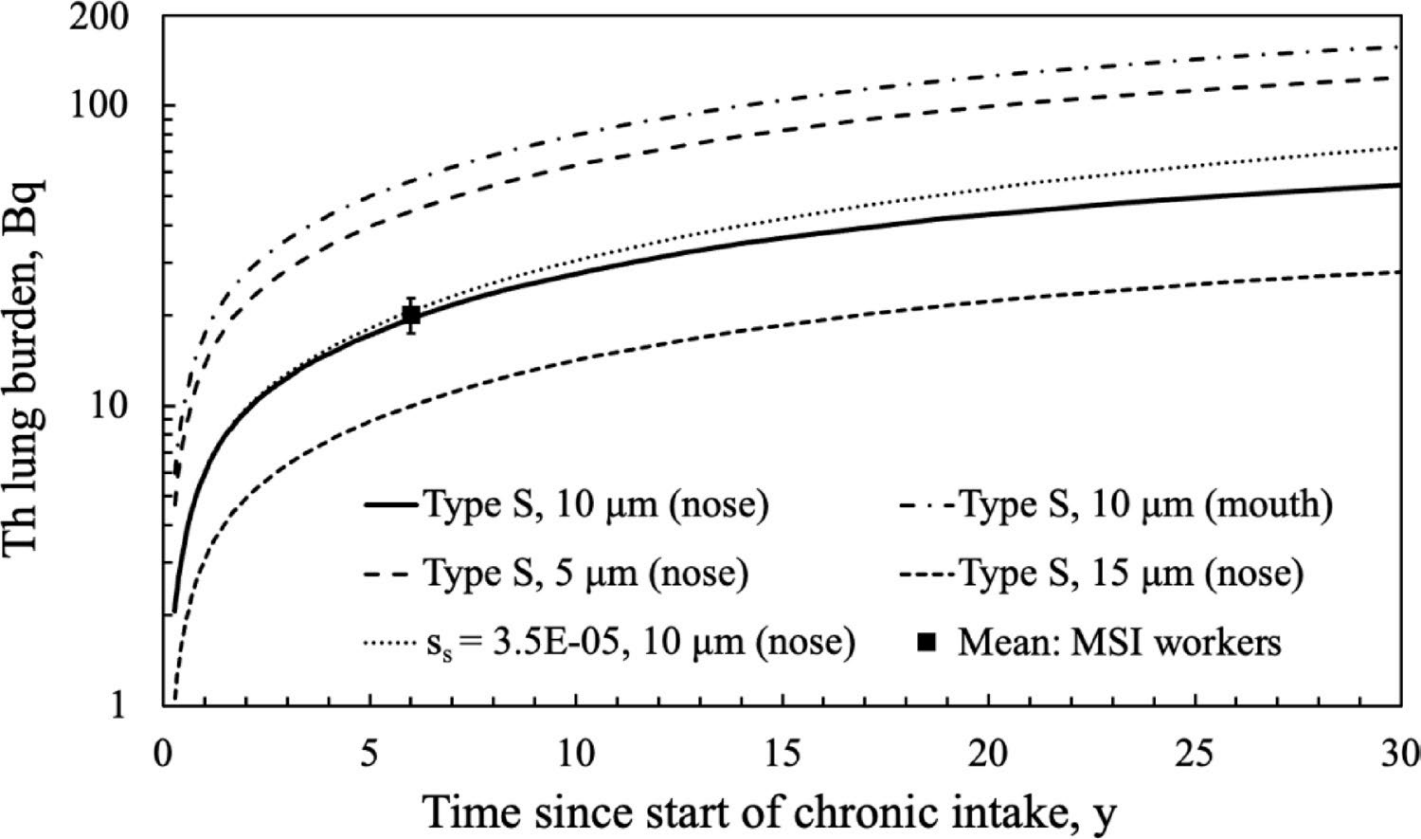
Thorium lung burdens for inhalation of 1 Bq·d^− 1^ thorium as a function of intake duration and different model input parameters. The group mean result from past thoron-in-breath testing of 40 MSI workers is also shown, with the error bar indicating the 95% confidence interval

The reinterpretation of the urine bioassay and TIB datasets against current biokinetic models included an analysis of the impact of altering default model parameter values, such as particle size and mode of breathing. In this study, thorium retention and excretion curves derived from Taurus were used to determine the daily thorium intake predicted from the mean bioassay results for the test cohort for comparison with the cohort’s mean recorded daily intake from PAS. For this study, only bioassay data for workers employed in the industry since 1985 were analysed owing to the existence of reasonable quality PAS data and employment records. Table 2 summarises the data for this sub-cohort of workers and Figs. 1 and 2 illustrate the processes used for both datasets.

**Table 2:** Summary bioassay data used in PAS comparison and sensitivity analyses

Bioassay dataset	No. workers	Parameter	Range	Mean ± SD (GSD)
Urinary thorium	26	^232^Th excretion^a^, mBq d^− 1^	0.023–1.6	0.34 ± 0.31 (2.7)
Thoron-in-breath	40	^232^Th lung burden^b^, Bq	8.4–40	20 ± 8.6 (1.5)

a Based on daily urinary volume of 1.9 litres for an 85 kg male worker.b Using a thoron exhalation rate of 3.7% to convert the breath measurement to thorium lung burden.

The method of applying Taurus to each bioassay dataset has been described in individual studies (Hewson et al. 2025b, d, 2026b). In summary, the Taurus output for a particular chronic intake scenario was exported to Excel and polynomial functions were fitted to the excretion and retention curves. The worker’s daily thorium intake was estimated by comparing the worker’s measured bioassay value with the predicted excretion or retention at 1 Bq·d^− 1^ for the intake duration at the time of the bioassay test.

Analysis was performed for the full dataset from the urine and TIB bioassay tests, as well as for the subset of workers who commenced work in the MSI from 1985 onwards (i.e., when PAS intakes were considered more reliable). For the urine and TIB bioassay tests, the reference chronic intake scenario, as depicted by the solid lines in Figs. 1 and 2, was based on the following assumptions:

- NORM radionuclides – thorium series in secular equilibrium,
- dust solubility - Type S (slow),
- activity median aerodynamic diameter (AMAD) − 10 μm.
- work activity - nose-breathing male worker doing heavy work.

Type S for NORM dust intake is recommended by the IAEA (2018) and supported by research into monazite dust solubility in simulated lung fluids (Twining et al. 1993). The ICRP default AMAD is 5 μm, however AMADs of 10 μm or larger have been measured using personal cascade impactors on mineral sand workers (Hewson et al. 2025e). Default absorption parameters for Type S thorium compounds, and the breathing rate for heavy work of 1.7 m^3^ h^− 1^, were obtained from ICRP Publications 137 (2017) and 130 (2015), respectively.

Sensitivity analyses were performed via Taurus to assess the impact of changing the particle size from 10 μm to 5 μm and 15 μm AMAD aerosols, the mode of breathing from the nose to the mouth, and a one-third reduction in the slow dissolution rate (s_s_) parameter (i.e., deposited material cleared to blood). The choice of s_s_ is expected to significantly influence thorium excretion and retention (Hewson et al. 2025b).

## PAS protocols and exposure data

Regulatory agency guidelines on airborne radioactivity sampling and dose assessment (GWA, 2010a, 2023), together with a study (Hewson et al. 2025e) analysing potential issues associated with estimating the intake of NORM dust via PAS, were reviewed to summarise issues related to PAS analysis protocols and strategies. Historical PAS measurements for airborne radioactivity concentrations recorded for MSI workers from 1999 to 2009 were extracted from archived Western Australian Government (Department of Local Government, Industry Regulation and Safety - LGIRS) databases to investigate inter-worker variability, appropriateness of SEG assignments, and dust loadings on PAS filters. Owing to a change in regulatory policy, detailed PAS data post-2009 were not held by the regulatory agency but were instead maintained by individual mining companies. However, the findings from this study are anticipated to be generally applicable to contemporary MSI operations because mineral processing technology and regulator-approved PAS methodology have remained the same.

The archived PAS “dust” exposure database (GWA, 2010b) included the date of measurement, worker ID code, SEG code, type of dust sampler, sampling time, volume of air sampled, PAS filter weights before and after sampling, alpha particle detector efficiency, and sample and background alpha particle count rates. The archived records for the most recent 5-year period (2005–2009) were subsequently exported to Excel for statistical analysis, and the exposure dataset (*n* = 2038 PAS records) for the site with the highest historical monazite production was selected for detailed review. This site was denoted “Company C”, and the archived PAS data presented as high-quality and appeared to have been properly maintained and verified by the company prior to submission to the regulator. Differences between an individual’s PAS alpha activity concentration distribution and the SEG distribution were evaluated via Excel using a two-tailed t-test (α = 0.05) after log transformation of the data. All the PAS measurements for the 2005–2009 period were related to the inhalable dust fraction and were collected via a “7-hole” sampler (Standards Australia, 2009), as specifically recommended in local regulatory guidance (GWA, 2010). The inhalable dust concentrations for the treatment plant operator SEG and maintainer SEG were relatively stable over this 5-year period, with mean concentrations of 3.8 ± 3.7 mg·m^− 3^ (*n* = 835 PAS) and 2.4 ± 3.2 (*n* = 245 PAS) mg·m^− 3^, respectively.

## Results

The PAS results listed below do not account for the use of respiratory protective equipment by workers; therefore, it may be expected that the actual PAS-derived intakes for some workers were less than those recorded and reported to the regulator at the time.

### Ratio of estimated bioassay intake to recorded intake via PAS

Table 3 shows a consolidated comparison of the recorded and recently revised bioassay-derived daily intakes of thorium for MSI workers, extracted from previously published studies (Hewson et al. 2025b, d, 2026b). On average and across the cohort of workers tested in each study, bioassay-derived intakes were consistently greater than those recorded on the basis of PAS.

**Fig. 3:**
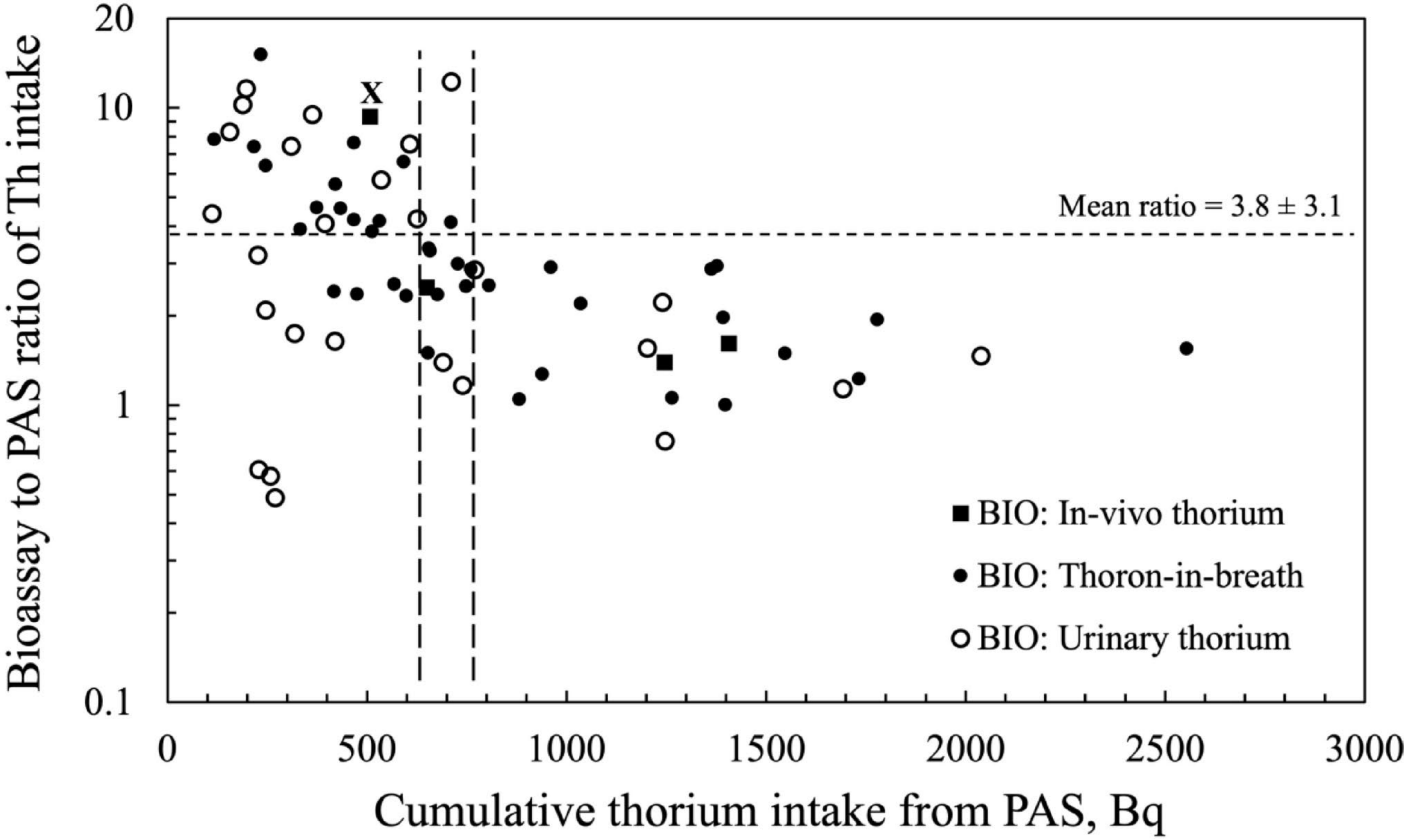
Ratio of bioassay-derived and PAS-derived daily thorium intake for mineral sand workers employed since 1985 as a function of cumulative intake. Bioassay estimates calculated for 10 μm AMAD, Type S aerosols and a nose-breathing male worker

Figure 3 shows the ratio of bioassay-derived to PAS-derived daily intake of thorium for the sub-cohort of workers employed since 1985. Three sets of bioassay-to-PAS ratios were plotted: (1) in vivo lung counting (*n* = 4), (2) TIB testing (*n* = 40), and (3) urine bioassay (*n* = 26).

The mean ratios for the three types of bioassay tests for this sub-cohort were similar (3.7–4.2), indicating that the recorded intakes determined by PAS were likely understated for many workers on the basis of their bioassay test results. 62% of the 70 workers tested had a ratio > 2, and an order of magnitude difference in ratios was noted among workers with a similar recorded cumulative intake based on PAS. For example, workers who had a PAS estimate of cumulative thorium intake of 700 ± 70 Bq, had bioassay estimates of intake that varied from 1 to 10 times that of PAS (denoted by dashed lines in Fig. 3). The worker denoted “**X**” in Fig. 3 had a recorded cumulative thorium intake of 510 Bq derived from SEG PAS measurements; however, analysis via Taurus indicated that the worker’s in vivo thorium lung burden of 41 ± 20 Bq after seven years of intake corresponds to a cumulative intake of 4,600 Bq, which is nine-times greater.

**Table 3:** Comparisons of estimated daily thorium intake from PAS exposure records and revised bioassay results from mineral sand workers

Type of bioassay	No. of workers tested	Chronic intake period, y	Estimated mean daily thorium intake^a^, Bq·d^− 1^		Mean ratio^a, b^ - bioassay: PAS
			Revised bioassay^c^	Recorded (PAS)	
Faeces (10 d)	2	13–17	3.2 ± 0.014	1.3	2.6
Urine^d^ (24-h)	26	1.0–6.5	1.8 ± 1.7 (2.8)	0.51 ± 0.29 (1.7)	4.2 ± 3.7 (2.7)
In vivo (lung)^d^	4	6.2–8.1	0.99 ± 0.54 (1.6)	0.38 ± 0.20 (1.8)	3.7 ± 3.8 (2.4)
Thoron-in-breath^d, e^	40	3.0–8.5	0.99 ± 0.40 (1.5)	0.35 ± 0.19 (1.8)	3.7 ± 2.7 (1.9)

a Mean ± standard deviation; geometric standard deviation (GSD) in brackets.b Mean ratio of the individual measurement ratios.c Revised bioassay intake figure on the basis of reanalysis of the original data via current ICRP models.d Workers employed since 1985 and where the recorded intake assessment was based only on PAS.e A further 63 workers employed since 1985 were assessed as being below the MDL of the TIB method (lung burden of ~9 Bq). The mean intake period for these workers was 5.0 y, and the PAS-derived mean daily thorium intake was substantially lower at 0.15 ± 0.14 Bq·d-1.

## Impact of changes to model input parameters

Table 4 summarises the output of the sensitivity analysis resulting from the changes in the model input parameters as illustrated in Figs. 1 and 2. The importance of understanding the material-specific parameters for input into internal dosimetry models is highlighted by the variability in the predicted daily thorium intake in Table 4. For example, the sub-cohort of workers had predicted daily thorium intakes from urine bioassays varying from 0.62 to 3.2 Bq·d^− 1^ depending on the assumed aerosol characteristics and mode of breathing. These values contrast with the recorded mean daily PAS intake of 0.51 Bq·d^− 1^. Similar findings were observed in the TIB tests: 0.35–1.9 Bq·d^− 1^ vs. a mean PAS value of 0.35 Bq·d^− 1^.

**Table 4:** Impact of changes in model input parameters on the estimated mean daily thorium intake and annual dose for MSI workers employed since 1985

Bioassay type	Intake (inhalation) scenario	Est. mean thorium intake, Bq·d^− 1^	Est. dose^a^, mSv⋅y^− 1^
Urinary thorium (*n* = 26)	Using recorded PAS mean for MSI workers	0.51 ± 0.29	10
	1) 10 μm AMAD, nose breather (reference)^b^	1.8 ± 1.7	35
	2) 5 μm AMAD, nose breather	0.89 ± 0.81	32
	3) 15 μm AMAD, nose breather	2.9 ± 2.6	36
	4) 10 μm AMAD, mouth breather	0.62 ± 0.56	29
	5) Slow dissolution rate, s_s_ = 3.5E-05 d^− 1^	3.2 ± 2.9	84
Thoron-in-breath (*n* = 40)	Using recorded PAS mean for MSI workers	0.35 ± 0.19	6.9
	1) 10 μm AMAD, nose breather (reference)^b^	0.99 ± 0.54	20
	2) 5 μm AMAD, nose breather	0.44 ± 0.18	16
	3) 15 μm AMAD, nose breather	1.9 ± 0.78	24
	4) 10 μm AMAD, mouth breather	0.35 ± 0.14	17
	5) Slow dissolution rate, s_s_ = 3.5E-05 d^− 1^	0.95 ± 0.38	25

a Thorium series dose coefficients, in mSv·Bq-1, derived from Taurus for the five scenarios listed are as follows (in order): 0.054 (also used for PAS), 0.10, 0.034, 0.13 and 0.072. The coefficients assume secular equilibrium in the thorium decay series.b The reference intake scenario refers to the intake of a Type S, 10 µm AMAD thorium aerosol by a nose-breathing male worker.

## SEG assignment evaluation

Table 5 summarises the airborne alpha activity concentration data (*n* = 335) reported by Company C for the period from 2005 to 2006. This period was selected because three workers had 24 or more PAS results, thus allowing a statistical comparison with the SEG average data. The data were for the SEG denoted “dry-plant operators” and it was assumed that the workers in this SEG performed similar operational tasks during their daily work shifts and had comparable exposure profiles. A retrospective determination of the total analytical uncertainty (1σ) associated with the recorded (PAS) alpha activity concentrations across the dataset indicated a range of 11–51%, with the higher value corresponding to samples denoted as < MDL. For the dry-plant operator SEG, the average uncertainty was 14%, consisting of alpha counting (± 8%), equipment calibration (± 5%) and sample volume (± 10%). Only 2.1% of the PAS alpha activity measurements for this SEG were recorded to be < MDL.

The geometric mean (GM), geometric standard deviation (GSD), arithmetic mean (AM) and standard deviation (SD) are shown in Table 5. The GM has been included because local regulatory guidelines refer to the use of the GM in dose assessment calculations (GWA, 2023). The percentages of PAS filters with dust loadings > 0.30 mg·cm^-2^ are also shown because loadings above this level suggest the potential for significant understating (> 10%) of activity owing to alpha particle self-absorption within the mineral dust matrix (Hewson et al. 2026a).

**Table 5:** Summary of reported airborne alpha activity data for MSI dry-plant operators at Company C for the period 2005–2006^a^

SEG or Worker ID	No. PAS^b^	Alpha activity, mBq·m^− 3^		t-test probability^c^	% PAS > 0.30 mg·cm^− 2^
		GM (GSD)	AM ± SD		
SEG	335 (47)	230 (2.7)	350 ± 310	-	79%
-424	25	360 (2.2)	510 ± 566	0.015 (S)	84%
-375	25	160 (2.5)	220 ± 170	0.041 (S)	44%
-429	24	190 (2.1)	240 ± 150	0.22 (NS)	83%

a Data extracted from archived Western Australian Government exposure databases retained by LGIRS.b The number in parentheses is the number of individual workers sampled in the SEG.c Comparison of worker’s PAS results with the SEG results. S = significant (a = 0.05); NS = not significant

.

Some key points from Table 4 include the following:

- The GSD for the SEG of 2.7 indicated non-homogeneity in exposures within the SEG and a major impact of individual work practices on exposure variability or periodic nonroutine exposure episodes. Ideally, the SEG GSD should be < 2.5 for workers within the group to have a similar exposure profile. SEG GSDs ≥2.5 indicate highly variable exposures and possibly inappropriate worker assignment (AIOH, 2014). The mean PAS result for Worker 424, for example, was higher than the mean result for Worker 375 by a factor of 2.3, and the individual PAS distributions were significantly different (paired t-test probability = 0.0017).
- For two of the three workers listed (Workers 424 and 375), the difference between their individual PAS datasets and the SEG dataset was statistically significant. Comparisons between a worker’s annual mean and the SEG mean showed a range from 46% underestimation (Worker 424) to 59% overestimation (Worker 375) of individual intake.
- All workers listed were likely to be exposed to substantially greater mean alpha activity concentrations given that a high percentage (44–88%) of their PAS filters had dust loadings of more than 0.30 mg·cm^-2^.

The data in Table 5 indicate that the use of the GM concentration in dose calculations can result in a significant under-statement of dose, especially when the SEG GSD is greater than 2. If the regulator’s approved intake protocols (GWA, 2023) were applied, Worker 424 would have been assigned the SEG GM airborne alpha activity concentration of 230 mBq·m^-3^; however, the worker’s individual PAS results (*n* = 25) indicated an AM alpha activity concentration of 510 mBq·m^-3^. This higher activity concentration results in a dose that is 2.2 times greater than that recorded for the worker at the time.

## Discussion

The observed differences between the PAS-recorded and bioassay-derived thorium intakes may be due to several factors including inappropriate model input parameters, potential dust sampler bias, the PAS monitoring schedule and strategy, high variability of exposure within SEGs, self-absorption of alpha particles during radiometric analysis of filtered dust samples, and not accounting for respiratory protection use.

### Model input parameters

The EURADOS IDEAS guidelines recommends sophisticated internal dosimetry evaluation using workplace-, individual- and material-specific information if internal doses are likely to be ≥6 mSv (Castellani et al. 2013). As noted in Table 4, estimated annual mean doses to the MSI worker cohort were above this criterion, indicating that use of alternate model input parameters is appropriate.

The model parameters of key importance in assessing intake from bioassay measurements are the particle size of the inhaled dust (AMAD), the solubility classification of dust (i.e., absorption into the blood), the particle density and shape, and the mode and rate of breathing.

For thorium and uranium series radionuclides associated with insoluble mineral dust matrices, such as mineral sand dust, the solubility classification is generally assumed to be Type S (slow absorption to the blood) and hence avid retention of dust in the lungs (Hewson et al. 2025a; IAEA, 2011). If the dust is more soluble in lung fluids (e.g., Type M), the dose would be much lower. (Hewson et al. 2025c).

#### Particle density and shape

Particle density and shape influence deposition in the respiratory tract and have the greatest influence on the deposition of smaller particle sizes, typically < 1 μm (ICRP 2015). Monazite has a density (~ 5.2 g·cm^− 3^) that is significantly greater than the ICRP default particle density of 3.0 g·cm^− 3^; however, it was recently shown that the inhalation dose coefficient for thorium was not significantly affected at particle sizes > 5 μm when a higher density was used for monazite dust (Hewson et al. 2025c).

#### Particle size (AMAD and GSD)

 The particle size distribution (AMAD and GSD) of workplace dust is important for two reasons: (1) aerosol AMAD and GSD determine the relative deposition of inhaled particles in various regions of the respiratory tract, and hence the inhalation dose coefficient, and (2) the distribution of dust particle sizes influences the sampling efficiency of the PAS device used. The ICRP recommends a default AMAD of 5 μm in the absence of material-specific data (ICRP 2015); however, typical measured AMADs in the MSI were ≥10 μm (Hewson et al. 2025e).

#### Mode of breathing

The mode of breathing (nose vs. mouth) also had a significant effect on the shape of thorium excretion (Fig. 1) and retention (Fig. 2) functions. The bioassay studies in this research adopted the ICRP default position of a nose breather but considered a higher rate of breathing (1.7 vs. 1.2 m^3^·h^− 1^) because of the more strenuous work activities associated with operators and maintainers of MSI process plants. Sensitivity analysis (Table 4) using Taurus revealed that similar doses were estimated from urine and TIB bioassay measurements, irrespective of the breathing mode or particle size. This occurred because the difference in the daily intake of thorium derived from either the excretion or retention functions (Figs. 1 and 2) was offset by the change in the inhalation dose coefficient. However, the mean estimated daily thorium intake between mouth and nose breathing was 2.9 and 2.8 times lower for the TIB and urine studies, respectively. While the intake values for mouth breathers aligned better with the PAS data, it is unrealistic to expect that all the workers tested were predominantly mouth breathers.

#### Slow dissolution rate to blood

One model parameter that was found to cause a material difference to the intake and dose estimated from urine bioassay was the slow dissolution rate. For example, a threefold change in the clearance half-time of thorium deposited in the lungs to the blood from 7,000 to 20,000 days results in twofold lower thorium excretion in urine (Fig. 1). Consequently, a much greater daily intake and estimated annual dose were associated with the same measured bioassay value (Table 4). However, justifying the application of different absorption parameters in the absence of material-specific data is difficult. Investigating this parameter, which the ICRP acknowledges has large uncertainty, could be a potential focus of future research.

### Dust sampler collection efficiency bias

Dust sampler performance is related to particle size and workplace environmental conditions such as air velocity (Kenny et al. 1997). The MSI has used a 7-hole dust sampler in PAS programs since 2000. This sampler, which is attached to the worker’s collar, collects dust and associated radioactivity on a 25 mm filter via seven equally spaced 4 mm inlet holes. The sampler is known to underestimate the inhalable dust concentration when used in workplace atmospheres with a prevalence of coarse particles with aerodynamic diameters > 10–15 μm (Kenny et al. 1997; Terry et al. 1998). A recent review of NORM dust sampling (Hewson et al. 2025e) recommended replacing the 7-hole sampler with a more efficient inhalable dust sampler that closely matches the ISO inhalability convention (ISO 1995). These authors concluded that the 7-hole sampler underestimated inhalable dust concentrations in the MSI, which is characterized by an AMAD of ≥10 μm, by at least twofold. The implication is that PAS-derived radionuclide intake is likely to be significantly understated for the period during which the 7-hole sampler has been in use (i.e., > 25 years), assuming that workplace dust and activity distributions (AMAD and GSD) have remained similar. In NORM industries with much smaller median dust particle sizes, this sampler bias effect is likely to be less important.

### Alpha particle self-absorption

Alpha particle self-absorption (i.e., alpha particles that do not escape large dust particles or a layer of dust deposited on the PAS filter) is a potential issue when analysing filtered dust collected from PAS via alpha particle counting (IAEA, 2018). A recent review of this topic (Hewson et al. 2026a) concluded that airborne alpha activity concentrations recorded from PAS measurements collected from the more highly exposed MSI workers from 2004 to 2009 were underreported by 30–50%. This conclusion was reached after each PAS measurement was adjusted via a trinomial correction formula for filter dust loading (DL) derived from a meta-analysis conducted by Barnett and Edwards (2022), presented as Eq. (1):

1 $$ \begin{gathered} \:\% \:{\mathrm{alpha}}\:{\text{self - absorption = 0}}.{\mathrm{283}} \times \:{\mathrm{DL}}^{{\mathrm{3}}} {\text{ - 4}}.{\mathrm{90}} \times \:{\mathrm{DL}}^{{\mathrm{2}}} \hfill \\ \,\,\,\,\,\,\,\,\,\,\,\,\,\,\,\,\,\,\,\,\,\,\,\,\,\,\,\,\,\,\,\,\,\,\,\,\,\,\,\,\,\,\,\,\,\,\,\,\,\,\,\,{\text{ + 30}}.{\mathrm{3}} \times \:{\mathrm{DL}}\left( {{\text{r2 }} = {\text{ }}0.{\mathrm{95}}} \right)~ \hfill \\ \end{gathered} $$

Alpha self-absorption was estimated at 10% at a filter dust loading of approximately 0.3 mg·cm^-2^ and increased to more than 50% at a dust loading of 3 mg·cm^-2^. No evidence was found that self-absorption correction factors had been applied to past PAS data in the MSI, even though 79% of all PAS measurements on dry-plant operators between 2005 and 2006 had filter dust loadings greater than 0.3 mg·cm^-2^ (refer to Table 5). For example, Worker 424 had an average filter dust loading of 0.80 mg·cm^-2^ across 25 PAS measurements in 2005–2006. If Eq. (1) is applied to the dust loading recorded for each PAS measurement conducted on the worker, the annual mean alpha activity increases to 710 mBq·m^-3^, which is 3.1 times greater than the SEG GM alpha activity assigned to the worker for the 2005–2006 period.

Updated industry guidance is recommended to ensure that self-absorption effects are properly accounted for during the radiometric analysis of PAS filters in NORM industries where dust levels may be significant.

### Appropriateness of similar exposure group (SEG) assignments

The internal dose assessment protocol for Western Australian NORM-exposed workers relies on the measurement of the total alpha activity concentration obtained from PAS filters collected from workers assigned to defined SEGs. Table 5 shows that the annual mean airborne alpha activity concentrations measured on individual dry-plant operators can be significantly different from the annual mean value for the SEG to which they are assigned. Hence, the internal dose recorded for a worker may not be representative of the actual dose received on the basis of the worker’s individual PAS results. The representativeness of the annual mean activity concentration assigned to all workers in a SEG may explain some of the inter-worker variability noted in Fig. 3.

Recently, Ralph et al. (2024) reported an 81% reduction in the number of PAS samples per designated radiation worker in the Western Australian NORM industry between 1993 and 2023, from 1.4 to 0.27 samples per worker per year. Therefore, many workers were not sampled during the annual reporting periods, further highlighting the importance of appropriate SEG classifications and worker assignment to SEGs. The use of the SEG mean for a worker within the SEG is acceptable if the exposure is not highly variable and is a small fraction of the derived air concentration limits or regulator-defined action levels. However, as exposure approaches established action levels, differences in individual work and hygiene practices become important, and greater emphasis is needed on a statistically appropriate monitoring strategy. A review of the use of SEGs and sampling quotas by industry to confirm statistical validity (e.g., by reviewing log-normal distribution parameters and SEG definitions vis-à-vis current work activities) seems timely and prudent. Establishing SEG-specific average worker breathing rates on the basis of the extent of activity (e.g., light work @ 1.2 m^3^·h^-1^ vs. heavy work @ 1.7 m^3^·h^-1^) should also improve the accuracy of intake assessments.

Another issue associated with the current SEG methodology is that regulatory guidance (GWA, 2023) refers to the use of the geometric mean in intake calculations. The arithmetic mean is the appropriate statistical parameter for determining the cumulative exposure, such as annual intake of radionuclides, where a linear no-threshold dose response model is assumed (Crump 1998; Sexias et al., 1988). The geometric mean understates the actual intake by a factor that depends on the GSD for the SEG distribution of activity concentrations. As shown in Table 5, the GSD for the dry-plant operator SEG was 2.7, indicating highly variable daily exposures.

### Accounting for use of respiratory protection

The differences noted between the bioassay-derived and PAS-derived intakes for the MSI worker cohort are likely to be even greater than those indicated in Table 3 because the recorded PAS intake for each worker did not account for any potential use of respiratory protection. For long-term workers, the impact may be minimal because respirator use was uncommon or not rigorously enforced prior to the mid-1980s (Hewson and Terry 1995). However, for workers who commenced employment post-1985, the impact could be very significant, depending upon individual worker diligence in wearing respirators during dusty work tasks and the effectiveness of supervision. A study conducted during the early 1990s investigated workplace protection factors for MSI workers who intermittently wore half-face filter cartridge respirators for specific locations and tasks during work shifts (Hewson and Ralph 1992). The authors concluded that a workplace protection factor of at least 3.5 (attained in over 90% of the tests) could be applied to the average ambient airborne radioactivity concentration to obtain a conservative estimate of the actual inhaled radioactivity concentration.

The extent of respiratory protection use by workers was likely a further contributing factor to the large variability observed between the bioassay and PAS intakes for workers with similar cumulative PAS-derived intakes, as illustrated in Fig. 3.

### Study limitations and model uncertainties

The historical PAS and bioassay datasets used in this study will have limitations when considering the generalisation of findings and impact on contemporary NORM industries. Current MSI workers are exposed to approximately order of magnitude lower mean airborne alpha activity concentrations (Ralph et al. 2024), and consequently lower daily thorium intakes, than those workers who were employed since 1985 and who participated in past bioassay studies. Lower thorium intake will affect the feasibility of some bioassay tests for current workers and will require the use of sensitive analytical methods.

The current regulator-approved PAS methodology (GWA, 2023) has remained essentially the same since it was first implemented in the late 1980s (Hewson 1990), including random sampling, SEG definitions and worker allocation to SEGs. The fundamental limitations associated with sampler bias, SEG assignment uncertainty, and alpha self-absorption effects, will remain relevant wherever airborne alpha activity is monitored using PAS. Accounting for the impact of the use of respiratory protection is problematic as detailed information on type and frequency of use was not recorded. However, any protective effect exacerbates the differences between PAS and bioassay noted in this study.

Some statistical comparisons (e.g., Table 5) were based on relatively small worker-specific datasets and should therefore be interpreted cautiously, particularly given the lognormal nature of occupational exposure data and the potential influence of high-exposure outliers.

The bioassay measurements have uncertainties associated with the representativeness of the collected sample and subsequent radiological analysis. The EURADOS IDEAS guidelines indicated typical scattering factors (uncertainty) of 1.2, 1.6 and 2 for in vivo measurements, 24-hour urine samples, and 72-hour faecal samples, respectively (Castellani et al. 2013). There is also uncertainty associated with environmental intake of thorium by workers, although the bioassay cohorts were selected on the basis of significant occupational thorium-bearing dust intake.

Bioassay-derived dose estimates may provide a more direct indication of systemic intake than PAS-derived estimates, although both approaches depend on dose coefficients that are associated with substantial uncertainty and variability (Breustedt, 2018; European Commission, 2018; Paquet 2022). The ICRP dosimetric and biokinetic models are based on simplifications of human physiology and anatomy and an approximation of radionuclide distribution and radiation-matter interaction in the human body (European Commission, 2018). There will also be individual variability from the reference person used in the models and differences between assumed and actual workplace exposure conditions.

In this study, the bioassay-derived estimates, as listed in Table 4, appeared less sensitive to plausible variations in model parameters than the PAS-derived estimates. PAS-derived doses are very sensitive to changes in model parameters, such as AMAD, solubility, and mode and rate of breathing. The estimated doses in this study were based on the measured radiological, physical and chemical properties of the mineral dust matrix and the assumption of chronic intake. Assessment of other sources of uncertainty and variability were outside the scope of this study, but it is important that these factors be considered when reconstructing doses for epidemiological or compensation purposes (Toohey, 2008).

### Summary of PAS vs. bioassay for assessing NORM dust intake

Despite the potentially significant differences observed between PAS and bioassays across several studies in the MSI, both methods have a role in monitoring operations where exposure to NORM dust may constitute a significant exposure pathway. Depending on the extent of NORM exposure, the two methods are generally complementary. PAS offers operational feasibility and timely feedback on exposure data, whereas bioassays provide direct evidence of internal uptake by individual workers. Table 6 summarises the relative advantages and disadvantages of each method applicable to NORM dust intake. Given the potential understatement of intake from PAS as outlined above, bioassay tests may be feasible for selected cohorts of NORM-exposed workers.

The IAEA (2018) recommends the use of PAS as a primary monitoring tool in routine NORM operations. PAS is the preferred method where intake levels, and hence doses, are anticipated to be significantly below regulatory limits or reference levels (e.g., ~ 1–5 mSv·y^− 1^). However, appropriate PAS protocols and aerosol-specific parameter values should be used to reduce the potential for underestimation or overestimation of the intake and dose. Bioassay monitoring should be considered when chronic intake is high or when abnormal intake is suspected, and higher accuracy or individual dose confirmation is needed. The ICRP (2015) suggests that occasional bioassay measurements may be prudent for radionuclides that are avidly retained in the body (e.g., thorium and uranium) to confirm the absence of activity build-up within the body. The implementation of bioassay monitoring by industry should be supported by appropriate regulatory guidance to ensure a consistent methodology is used for interpreting data, such as the frameworks published by the European IDEAS Guidelines (Castellani et al. 2013) and International Organisation for Standardisation (ISO 2025). Guidance for bioassay tests specifically targeting the excretion or retention of thorium and uranium series radionuclides from the mining and processing of NORM-bearing minerals should be considered.

**Table 6:** Summary of PAS vs. bioassay for assessing NORM dust intake

Feature	PAS	Bioassay monitoring
Type of measurement	Airborne radioactivity concentration, typically total alpha activity.	Internal radionuclide retention (in vivo) or excretion (in vitro).
Exposure pathway	Inhalation only.	Inhalation, ingestion, dermal.
Reflects actual intake?	Indirect. Relies on several assumptions re: aerosol characteristics and efficient sampler collection.	Direct. Reduces uncertainty due to particle size, breathing rate, sampler efficiency, and individual metabolism.
Detection limits^a^	2–20 mBq·d^− 1^ Th or U (derived from total alpha counting).	In vivo: 10–30 Bq (lung) In vitro: 0.002–0.01 mBq·d^− 1^
Cost and practicality	Low-to-moderate. Operational simplicity and convenience.	High cost and detection limits may be inadequate.
Exposure feedback	Quick.	Slow (often retrospective).
Application	Group (SEG) monitoring, regulatory compliance.	Investigations, dose confirmation, high-risk workers, multiple intake routes.
Worker acceptance	Tolerable, non-invasive.	Moderate to high intolerance (depending on type of sample collected). Invasive.
Key limitation	Sampler bias re: aerosol fraction collected. Infrequent sampling may miss key exposure events. Alpha self-absorption correction needed.	Logistically complex, costlier, and requires internal dosimetry software to conduct sophisticated evaluations and interpret results.

a The detection limits are listed for Th and U as parents of naturally occurring decay chains. In vivo monitoring involves measuring the gamma-ray emissions of a daughter radionuclide and requires assumptions regarding decay chain equilibrium. In vitro monitoring refers to the measurement of urinary Th and U excretion via inductively coupled plasma–mass spectroscopy (ICP-MS).

## Conclusions

By integrating findings from earlier investigations, this study indicates that historical PAS-derived thorium intakes, and hence doses, for MSI workers were likely underreported. Across all the bioassay methods studied, the intake estimates were 2.6–4.2 times greater than those derived from PAS measurements. The differences between the bioassays and PAS were attributed to multiple factors, including the following:

- Uncertainties in biokinetic model intake parameters, particularly aerosol size and solubility and mode and rate of breathing.
- Dust sampler collection efficiency bias, especially with the continued use of the 7-hole sampler in coarse dust environments.
- Underreporting of airborne activity owing to neglect of alpha particle self-absorption when dust loadings on filters exceed established thresholds.
- The variability of inter-worker exposure within SEGs, infrequent sampling within an SEG and the use of inappropriate exposure parameters.

The implications of underestimation of intakes by PAS are particularly important in the event of future health studies, where such intakes are used for the basis of worker dose reconstruction. Although PAS remains a practical and necessary tool for operational monitoring and regulatory compliance, it may not reliably represent true worker intake in many NORM dust exposure situations unless appropriate correction factors are applied to the PAS data. Therefore, we recommend the following:

- Update PAS protocols, including the adoption of appropriate inhalable dust samplers, correction for alpha particle self-absorption on the basis of PAS filter dust loading, and SEG-specific breathing rates.
- Review the statistical validity of current SEG definitions and sampling frequency and use the arithmetic mean rather than the geometric mean airborne activity concentration for cumulative intake assessment.
- Incorporate targeted bioassay monitoring where feasible, especially for designated high-exposure risk NORM workers.
- Define protocols for the use of bioassay monitoring for dose assessment of NORM workers under regulatory guidance, including criteria for the implementation and use of aerosol-specific parameters in internal dosimetry models, together with methods to account for uncertainty and variability.

These recommendations can increase confidence in dose assessments for workers with the potential to inhale NORM dust and provide a more robust and protective monitoring regime for workers.

## Data Availability

The data underlying this article are available in the article and from publicly available reports and articles cited therein. The SEG PAS data used in this study are contained within confidential and archived databases held by the Western Australian Government (Department of Local Government, Industry Regulation, and Safety). These regulatory monitoring data are not publicly available.
